# Clinical and Epidemiological Characteristics of Stenotrophomonas maltophilia Associated Lower Respiratory Tract Infections in Qatar: A Retrospective Study

**DOI:** 10.7759/cureus.23263

**Published:** 2022-03-17

**Authors:** Arun P Nair, Sreethish Sasi, Muna Al Maslamani, Abdullatif Al-khal, Kadavil Chacko, Anand Deshmukh, Mohammed Abukhattab

**Affiliations:** 1 Infectious Diseases, Hamad Medical Corporation, Doha, QAT; 2 Infectious Disease, University Hospitals Coventry and Warwickshire NHS Trust, Coventry, GBR

**Keywords:** trimethoprim-sulfamethoxazole, levofloxacin, aeromonas, stenotrophomonas, gram-negative

## Abstract

Background

*Stenotrophomonas maltophilia* is a rapidly emerging nosocomial pathogen with intrinsic or acquired resistance mechanisms to several antibiotic classes. It can cause life-threatening opportunistic pneumonia, particularly among hospitalized patients. Incidence of infections by *S. maltophilia* has been reported as 0.07-0.4% of hospital discharges, but its mortality is 20 -60%. This is the first study from Qatar indexing the clinical and epidemiological characteristics and antibiotic susceptibility of *S. maltophilia.*

Materials and methods

This retrospective descriptive epidemiological study was conducted in 6 tertiary care hospitals under Hamad Medical Corporation in Doha, Qatar, analyzing inpatient respiratory isolates of *S. maltophilia *during 2016-17. Out-patients, children below 14 years, and non-respiratory samples except blood cultures in patients with pneumonia were excluded. Clinical records were reviewed to identify possible risk factors. Infection and colonization were identified using the Centers for Disease Control and Prevention (CDC) algorithm for clinically defined pneumonia and statistically analyzed using the chi-square test and Pearson's correlation.

Results

*S. maltophilia* was isolated from 2.07% (317/15312) of all respiratory samples received in the microbiology lab during our study period. Three hundred seventeen patients studied had a mean age of 60 ± 20 years, and 68% were men. Most of the isolates were from sputum (179), followed by tracheal aspirate (82) and bronchoscopy (42). Fourteen blood culture samples from patients diagnosed with pneumonia were also included. 67% were hospitalized for more than two weeks, 39.1% were on mechanical ventilators, and 88% had received a broad-spectrum antibiotic before the event. 29.1% were deemed to have an infection and 70.9% colonization. Incidence of infection in those with Charlson’s Co-morbidity Index (CCI) ≥ 3 was 36.5% compared to 24.2% in those with CCI < 3 (Relative Risk (RR)=1.52; 95% CI: 1.04,2.18; p=0.01). Patients with recent chemotherapy, immunosuppressant, or steroid use had a significantly higher infection risk than those without (69.2% v/s 23.3% RR=2.96; 95% CI:2.2,3.9; p<0.005). The most common symptoms in patients with infection were fever (96%) and expectoration (61.9%). The most common radiological finding was lobar consolidation (71.6%). Mean CRP and procalcitonin were 106.5±15.5 mg/l and 12.3 ± 14 ng/ml. Overall mortality was 16.3%. Patients on mechanical ventilator with IBMP-10 score ≥ 2 had 22.8% mortality compared to 5.7% in those with score < 2 (RR=3.9;95%CI:0.9,16.6; p<0.015). As per The US Clinical and Laboratory Standards Institute (CSLI) breakpoint values, Trimethoprim-Sulfamethoxazole (TMP-SMX) showed the highest sensitivity (97.8%), followed by levofloxacin (71.6%). 0.3% of samples were pan-drug resistant.

Conclusions

*S. maltophilia* is a frequent nosocomial colonizer, but it can cause nosocomial pneumonia in almost one-third of cases, specifically in immunocompromised and patients with CCI ≥ 3 with a high risk of mortality due to ventilator-associated pneumonia (VAP) in those with IBMP-10 ≥ 2. Prolonged hospital stay is a risk factor for colonization by *S. maltophilia,* while recent chemotherapy, immunosuppressant, or steroid use are risk factors for hospital-acquired pneumonia due to *S. maltophilia.* TMP-SMX and levofloxacin are the only reliable agents for monotherapy of respiratory infections due to S. maltophilia in Qatar.

## Introduction

*Stenotrophomonas maltophilia* is a ubiquitous, motile, free-living, aerobic, non-fermenting bacillus [1] that has intrinsic or acquired resistance mechanisms to several antibiotic classes. It was initially known as Bacterium bookeri and later as Pseudomonas maltophilia and Xanthomonas maltophilia. Biochemically, it is oxidase negative, catalase-positive, non-Lactose, and non-glucose fermenter. They appear as pale-yellow or lavender-green color colonies in blood agar with a typical ammonia-like odor [2]. Resistance to beta-lactams is conferred by two inducible beta-lactamases, a zinc-containing penicillinase (L1) and a cephalosporinase (L2) [3,4]. An aminoglycoside acetyl-transferase confers resistance to aminoglycoside antibiotics [5.6]. There are discrepancies in the optimal method for testing in vitro susceptibility [7]. The US Clinical and Laboratory Standards Institute (CSLI) has published disc-diffusion minimal inhibitory concentration (MIC) breakpoints for trimethoprim-sulfamethoxazole, minocycline, and levofloxacin and broth dilution MIC breakpoints for ticarcillin-clavulanic acid, ceftazidime, minocycline, levofloxacin, and chloramphenicol [8]. E-test may be used to evaluate ceftazidime, minocycline, and chloramphenicol susceptibility. There are no MIC interpretive criteria for tetracycline or tigecycline, although susceptibility to tigecycline is assumed using interpretive criteria for Enterobacteriaceae [9]. In contrast, the European Committee on Antimicrobial Susceptibility Testing (EUCAST) has only published breakpoint criteria for trimethoprim-sulfamethoxazole

S. maltophilia can cause respiratory tract infection, bacteremia, urinary tract infection, native or prosthetic valve endocarditis, meningitis, endophthalmitis, or keratitis in contact lens wearers, sinusitis, myositis, bone or joint infections, liver abscess, and peritonitis [10]. S. maltophilia pneumonia is usually hospital-acquired and frequently occurs in mechanically ventilated patients [11]. Compared with pulmonary colonization, infection is associated with underlying immunosuppression [11]. Risk factors for pulmonary diseases by S. maltophilia include prior broad-spectrum antibiotic therapy, presence of central lines, neutropenia, chemotherapy or corticosteroid use, prolonged hospitalization, recent surgery or trauma, admission to ICU, and use of mechanical ventilation or tracheostomy. It is found ubiquitously in hospital environments, including blood sampling tubes, central lines, contact lenses, disinfectants, nebulizers, ventilator circuits, and water sources [12].

Incidence of infections by S. maltophilia has been reported as 0.07-0.4% of hospital discharges [13]. S. maltophilia is distinguished by its armory of antibiotic resistance mechanisms, including beta-lactamases (for penicillin and cephalosporin), zinc-containing Metallo-beta-lactamases (lysing carbapenems), acetyltransferase (against aminoglycosides), and efflux pumps (against quinolones, carbapenems, aminoglycosides, and macrolides) [12]. S. maltophilia expresses many virulence factors such as biofilm, extracellular enzymes, and fimbriae, responsible for its pathogenicity as a nosocomial organism [12]. It colonizes the respiratory tract of hospitalized patients by biofilm formation. Differentiation between colonization and infection can be challenging due to the frequent isolation of other organisms from the same specimen. Moreover, the initially colonized patients may later develop an infection [13]. Although there is good evidence that S. maltophilia causes significant mortality in patients with nosocomial pneumonia [14], the significance of a positive respiratory isolate is much less evident in other clinical settings. A thorough search into the epidemiological characteristics regarding infection and colonization is warranted to ascertain the significance of respiratory isolates from patients. This is the first study from Qatar indexing the clinical and epidemiological characteristics and antibiotic susceptibility of S. maltophilia.

## Materials and methods

This retrospective study was conducted in 6 tertiary care hospitals under Hamad Medical Corporation in Qatar. These hospitals are Hamad General Hospital which is a 750-bedded hospital that offers trauma, emergency medicine, critical care, specialized surgery, specialized medicine, laboratory medicine, and radiology services; Rumaila Hospital, which is a 644-bed hospital offering rehabilitative services for disabled adults, and the elderly people; Women's Wellness and Research Centre which provides obstetrics, gynecology, neonatal care, emergency care and newborn screening services hosting 319 beds; National Center for Cancer Care and Research (NCCCR), an 86-bed research facility offering treatment for cancer patients; Heart Hospital, a cardiology specialty facility with 116 beds; and Al-Wakra Hospital, a 260-bedded general hospital. Ethical approval for this study was obtained from Medical Research Center at Hamad Medical Corporation (Approval Number: MRC- 17019/17).

A total of 504 isolates of S. maltophilia were reported by our microbiology lab during the study period. The isolates' identification was performed with conventional methods and matrix-assisted laser desorption/ionization - time-of-flight (MALDI-TOF) mass spectrometry. Antimicrobial susceptibility was detected using BD Phoenix™ automated identification and susceptibility testing system. Four hundred thirty-two consecutive non-duplicated samples were identified from this data. Out-patients, children below 14 years, and non-respiratory samples except blood cultures in patients with pneumonia were excluded. All respiratory samples from the remaining 317 patients were included in our cohort. Most of the isolates were from sputum (179), followed by tracheal aspirate (82) and bronchoscopy (42). Fourteen blood culture samples from patients diagnosed with pneumonia were also included. The subjects were divided into two groups, one infected by S. maltophilia and another colonized by S. maltophilia without clinical infection.

Infection and colonization were identified using the Specific Site Algorithms for Clinically Defined Pneumonia (PNU 1, 2, and 3) given by the Centers for Disease Control and Prevention (CDC, USA) [15]. The diagnosis of pneumonia requires the following criteria to be met. The patient should have at least one fever (>38.0°C), abnormal leucocyte count (≤4000/ >12,000 WBC/mm3), or altered mental status with no other recognized cause in adults >70 years. In addition, they should also have two among new onset of purulent sputum or change in the character of sputum, or increased respiratory secretions, or increased suctioning requirements, new-onset or worsening cough, or dyspnea, or tachypnea, rales or bronchial breath sounds, or worsening gas exchange (e.g., O2 desaturations or increasing PaO2/FiO2). Radiological features of pneumonia evidenced by two or more serial chest imaging showing new or progressive and persistent infiltrate, consolidation, or cavitation is also required. Hospital-acquired pneumonia (HAP), or nosocomial pneumonia, is a lower respiratory infection that was not incubating at the time of hospital admission and presents clinically two or more days after hospitalization. Pneumonia that presents sooner should be regarded as community­ acquired pneumonia. VAP refers to nosocomial pneumonia that develops among patients on ventilators. VAP presents more than 48 hours after endotracheal intubation [16,17]. Patients with fever (38 °C), cough, new or increased sputum production, rhonchi, wheezing, and a positive culture obtained by deep tracheal aspirate or bronchoscopy do not meet the criteria for pneumonia are considered to have tracheobronchitis or 'LRTI other than pneumonia.' These subjects were included in the 'infections' group (Group 2). The rest of the patients were considered colonizers (Group 1). Few cases met the criteria for infection but were not treated for S. maltophilia by infectious disease physicians. These patients had polymicrobial isolates, and the clinical symptoms were deemed to be caused by organisms other than S. maltophilia. Such cases were also included in the colonizer group. 

Demographic and clinical details were tabulated, including age, gender, clinical presentation, and comorbidities. Radiological and laboratory findings (white blood cells, C-reactive protein, procalcitonin, and blood urea nitrogen) at the time of isolation of S. maltophilia were obtained from electronic medical records. Details of antibiotic therapy and blood culture reports were collected to identify bacteremia due to S. maltophilia. The patients' clinical, laboratory, and radiological findings were correlated and compared to assess the significance of the S. maltophilia isolates. The descriptive analysis of the results was performed using Epi Info™ version 3.5.4. Comorbidities of the patients including age, myocardial infarction, congestive heart failure, peripheral vascular disease, cerebrovascular disease, dementia, chronic obstructive pulmonary disease (COPD), connective tissue disease, peptic ulcer disease, diabetes mellitus, chronic kidney disease, hemiplegia, leukemia, malignant lymphoma, solid tumor, liver disease, and AIDS were studied and expressed in terms of Charlson's Comorbidity Index (CCI). Outcomes of patients diagnosed with infection were expressed as 30-day all-cause mortality, length of hospital stay, length of intensive care unit (ICU) stay, and the number of days on a mechanical ventilator. IBMP-10 score (the presence of immunodeficiency, blood pressure < 90/60 mmHg, multi-lobar infiltrates on chest radiograph, platelet count <100,000/ µL and hospitalization >10 days before the onset of pneumonia) was calculated for patients with VAP and correlated with mortality.

Statistical analysis

Categorical and continuous values were expressed as appropriate as frequency (percentage) and mean ± SD or median and interquartile range (IQR). Descriptive statistics were used to summarize the patients' demographic, epidemiological, laboratory, and other clinical characteristics. Association between two or more qualitative variables (demographic, laboratory, and clinical features with Stenotrophomonas infection) were examined using the Chi-square test or Fisher exact test as appropriate. Quantitative data between two independent groups were analyzed using an unpaired 't-test. P values <0.05 were considered statistically significant.

## Results

S. maltophilia was isolated from 2.09 % (317/15312) of respiratory samples received in the microbiology lab during our study period (January 2016- January 2017). The three hundred and seventeen patients studied had a mean age of 60.5 ± 20 years, and 68% were men. Most of the isolates were from sputum (179). 67% of them were hospitalized for more than two weeks, and 88% had received a broad-spectrum antibiotic before the event. Based on the CDCs Specific Site Algorithms for Clinically Defined Pneumonia (PNU1, 2, and 3) [15], 70.9 % of the subjects were deemed colonized (Group 1) and 29.1 % infected (Group 2). Infection was in the form of pneumonia or tracheobronchitis. A comparison of demographic features, clinical characteristics, risk factors, and outcome measures of patients in the two groups is shown in Table 1.

**Table 1:** Comparison of Demographic features, Clinical Characteristics and Mortality of Patients with colonisation (group 1) and infection (group 2) caused by Stenotrophomonas maltophilia (n=317) a- Univariate Analysis, x±y indicates the mean with 95% confidence interval, b- New onset of purulent sputum or change in character of sputum, or increased respiratory secretions, or increased suctioning requirements, c- New onset or worsening cough, or dyspnoea, or tachypnea, d- Worsening gas exchange (e.g., O2 desaturations (e.g., PaO2/FiO2), e- Blood Urea Nitrogen, f- Before the date of isolation of organism in respiratory sample

Characteristics		Colonisation (Group 1, n = 225)	Infection (Group 2, n = 92)	p-value
Age^a^		59.52 ± 19.6	63.19 ± 23.67	0.03
Male Gender		146	69	0.04
Presenting Clinical Features	Fever	8	86	<0.01
	Sputum/ Secretions^b^	11	57	<0.01
	Breathing^c^	7	42	<0.01
	Gas Exchange^d^	5	32	<0.01
	Change in mental status	2	23	<0.01
Laboratory Findings	Leukopenia < 4,000/ Leucocytosis > 11000	63	35	0.041
	CRP > 10	190	87	0.005
	PROCALCITONIN (>0.5)	19	89	<0.01
	BUN^e ^> 7mmol/L	32	56	<0.01
Microbiology	Polymicrobial	179	18	<0.01
Radiological Findings	New onset Progressive infiltrate	0	28	-
	Cavitation	0	7	-
	Consolidation	0	65	-
	Pleural effusion	20	13	0.08
	Multi-lobar infiltrates	0	9	-
Severity Scores	Mean Charlson Comorbidity Index (CCI)^a^	2.25 ± 1.97	6.43 ± 3.49	<0.01
	Median IBMP-10 Score	0	2	-
Risk Factors	Hospitalization ≥ 2 weeks^f^	155	58	0.159
	Prior antibiotic use within 2 weeks^f^	201	78	0.13
	Stay in ICU^f^	36	23	0.03
	Recent chemotherapy, immunosuppressant or steroid use	12	27	<0.01
Outcomes	30-day all-cause mortality	2	12	<0.01

Mean age, mean Charlson's Co-morbidity Index (CCI), and median IBMP-10 score were higher for patients in group 2 (infection) than in group 1 (colonization). Incidence of infection in those with CCI ≥ 3 was 36.5% compared to 24.2% in those with CCI<3 (Relative Risk (RR) =1.52; 95% CI:1.04, 2.18; p = 0.01). Patients with recent chemotherapy, immunosuppressant or steroid use had a significantly higher risk of infection compared to those without [69.2% v/s 23.3% RR=2.96; 95% CI: 2.2, 3.9; p < 0.005)]. The most common symptoms in patients with infection were fever (96%) and new-onset purulent sputum (61.9%). The most common radiological finding was lobar consolidation (70.6%). Mean CRP and procalcitonin were 106.5±15.5 mg/l and 12.3±14 ng/ml.

79.5% (179/225) of the patients in group 1 had polymicrobial growth in their samples, and 41.3% (93/225) showed more than two organisms. Only 19.5% of the patients in group 2 had polymicrobial growth. Table 2 shows the various other organisms isolated along with S. maltophilia in polymicrobial samples.

**Table 2:** Other microorganisms isolated together S. maltophilia

		Colonisation (Group 1, n = 225)	Infection (Group 2, n = 92)
Gram-Negative	Pseudomonas aeruginosa	102	14
	Klebsiella sp.	45	4
	Acinetobacter sp.	21	3
	E. Coli	8	1
	Enterobacter sp.	13	2
	Acromobacter sp.	3	0
Gram-Positive	Staphylococcus aureus	25	2
	Enterococcus sp.	7	2
Candida sp.		49	9

**Figure 1:**
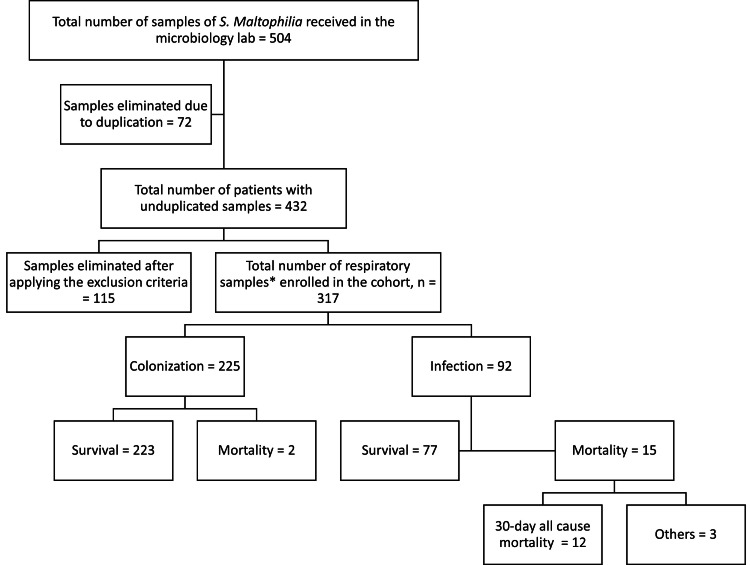
Flowchart showing the inclusion of patients with positive isolates of S. maltophilia reported from the microbiology lab and their final outcomes *For patients with respiratory symptoms, blood cultures were also included under respiratory isolates. Respiratory isolates included sputum, followed by endotracheal tube, tracheal aspirate, Bronchial wash, bronchoalveolar lavage, and blood

Pseudomonas aeruginosa is the organism that occurs most commonly with S. maltophilia. Overall mortality was 5.3 %. 30-day all-cause mortality in group 1 was two, and group 2 was 12. Three other patients in group 2 passed away during follow-up. 30-day all-cause mortality was significantly higher in group 2 compared to group 1 (RR=14.6; 95% CI: 3.3,64.2; p<0.005). Outcomes of 317 patients enrolled in the study are shown as a flowchart in Figure 1.

Patients in group 2 were further sub-divided into two groups, one for the survived and one for the deceased. A comparative study was done between the deceased and survived patients in the infected group to determine the risk factors that cause increased mortality in S. maltophilia infections (Table 3). Patients with IBMP-10 score ≥ 2 had 22.8% mortality compared to 5.7% in those with score <2 (RR=3.9; 95% CI: 0.9,16.6; p<0.015).

**Table 3:** Analysis of Outcomes of 92 patients who had infections due to S. maltophilia (Group 2) The patients who were diagnosed with S. maltophilia associated respiratory infections were compared on the basis of their outcome (Mortality v/s Survival). The various factors influencing mortality in these patients were identified a- Univariate Analysis, b – After diagnosis of respiratory infection associated to S. maltophilia x±y indicates the mean with 95% confidence interval

	Deceased (n=15)	Survived (n= 77)	p-value
Age	82.4 ± 4.3	59.4 ± 22.4	<0.01
Male Gender	12	57	0.33
Recent chemotherapy, immunosuppressant or steroid use	4	23	0.4
Haematological malignancy	2	4	0.15
Advanced cancer	1	3	0.3
Diabetes mellitus	8	22	0.03
Neutropenia (0.5 x 10^3^/µL)	6	9	0.08
Chronic Obstructive Pulmonary Disease (COPD)	3	6	0.09
Chronic kidney disease	4	14	0.23
Admission to intensive care unit^b^	13	6	<0.01
Need for vasopressors^b^	9	1	<0.01
Central venous catheter^b^	11	1	<0.01
Need for mechanical ventilation^b^	3	0	<0.01
S. maltophilia bloodstream infection	4	10	0.11
IBMP-10 score ≥ 2	13	44	0.015

**Figure 2:**
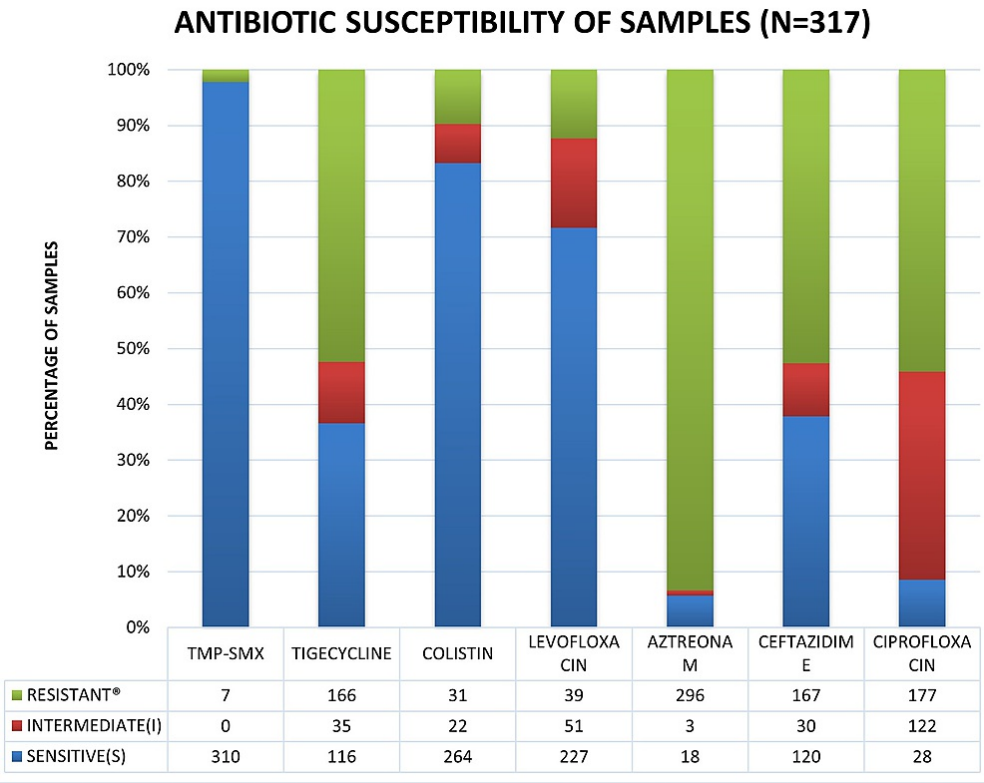
Graph showing the sensitivity S. maltophilia isolates from Qatar to common antibiotics Calculated by applying the Clinical and Laboratory Standards Institute (CLSI) break-point values for trimethoprim-sulfamethoxazole, minocycline, and levofloxacin; and broth dilution MIC breakpoints for ceftazidime. There are no MIC interpretive criteria for tigecycline, colistin, or aztreonam,  so susceptibility to them is assumed using interpretive criteria for Enterobacteriaceae. European Committee on Antimicrobial Susceptibility Testing (EUCAST) has only published breakpoint criteria for trimethoprim-sulfamethoxazole. Total number of samples, n = 317

The microbiological samples' sensitivities were calculated by applying the Clinical and Laboratory Standards Institute (CLSI) breakpoint values for trimethoprim-sulfamethoxazole, minocycline, and levofloxacin; and broth dilution MIC breakpoints for ceftazidime. There are no MIC interpretive criteria for tigecycline, colistin, or aztreonam, so their susceptibility is assumed using interpretive criteria for Enterobacteriaceae. European Committee on Antimicrobial Susceptibility Testing (EUCAST) has only published breakpoint criteria for trimethoprim-sulfamethoxazole. 97.8% of the samples were found to be sensitive to TMP-SMX. However, sensitivity to tigecycline (35%), ciprofloxacin (8.8%), and ceftazidime (39%) was low. The complete details of the sensitivity of S. maltophilia from Qatar are shown in Figure 2. Pan drug resistance was seen in 0.3% of the samples, resistant to all antibiotics mentioned above.

## Discussion

S. maltophilia is not an uncommon pathogen in respiratory samples from Qatar. International data show that bacteremia and hospital or ventilator-associated pneumonia caused by S. maltophilia results in significant mortality and morbidity, especially in severely immunocompromised and debilitated individuals [14,18]. Overall, mortality estimates range from 21 to 69 percent [19,20]. The actual mortality attributed to these infections when controlling for other variables is unclear. S. maltophilia can be readily identified on a culture of body fluids. Growth of S. maltophilia from sterile sites (such as blood, CSF, pleural fluid, or peritoneal fluid) should be interpreted to represent actual infection. However, since S. maltophilia can adhere to the mucosal surfaces of the upper airway and large bronchi and may colonize these areas without causing infection, it is important to differentiate colonization from actual infection due to S. maltophilia, particularly from respiratory isolates. In patients with clinical evidence of pneumonia (eg, new pulmonary infiltrate, decreased oxygenation, and fever and/or leukocytosis) [15-17], cultures growing S. maltophilia (with or without other concurrent respiratory pathogens) from respiratory sites should be interpreted to be consistent with true infection. In the absence of consolidation on chest radiography and other clinical signs of pulmonary infection, a positive respiratory tract isolate of Stenotrophomonas probably represents colonization rather than invasive disease. In a retrospective review of 92 patients presenting with acute respiratory symptoms and subsequently found to have respiratory cultures positive for S. maltophilia, there was no measurable impact of antibiotic therapy in the absence of chest radiograph consolidation [21]. However, in our study, patients with clinical symptoms of infection with positive S. maltophilia culture from respiratory samples were also considered actual infection rather than colonization. However, most isolates of S. maltophilia from the respiratory tract represent colonization rather than invasive disease. Only 29.1% of patients in our cohort with positive S. maltophilia samples could be diagnosed with clinical infection as per CDCs Specific Site Algorithms for Clinically Defined Pneumonia (PNU 1, 2, and 3) [15]. Patients who had S. maltophilia in their respiratory tract without meeting the criteria for infection were considered to be colonized by S. maltophilia. A major proportion (79.1%) of these patients had other organisms besides S. maltophilia. In our study, the commonest organisms found growing in samples with S. maltophilia were Pseudomonas aeruginosa, Klebsiella sp., and Staphylococcus aureus (Refer Table 2). The mortality rate was relatively high in the remaining 92 patients diagnosed with clinical infection. The 30-day all-cause mortality was 13% in the 'infection' group, which was lower than mortality rates from other international studies (21 to 69%) [19,20]. We believe that this lower mortality rate is because patients without radiological infiltrate or pneumonia, as per standard definitions [15-17], were also included in the 'infection' as tracheobronchitis. 

Even though infections due to S. maltophilia can occur in previously healthy patients [22,23], risk factors associated with S. maltophilia infection include admission to an intensive care unit (ICU), HIV infection, malignancy, cystic fibrosis, neutropenia, mechanical ventilation, central venous catheters, recent surgery, trauma, and previous therapy with broad-spectrum antibiotics [2,24-27]. The findings of our study suggested that patients with multiple debilitating illnesses are at higher risk of acquiring S. maltophilia related pneumonia. The risk of getting infected was directly proportional to the Charlson Co-morbidity Index (CCI). S. maltophilia pneumonia is usually hospital-acquired and frequently occurs in mechanically ventilated patients [28]. Clinical and radiographic findings are generally similar to those of other infectious causes of hospital-acquired pneumonia. In patients with hematologic malignancies, a syndrome of rapidly progressive and frequently fatal hemorrhagic pneumonia associated with S. maltophilia infection has been reported [29,30]. We found that diabetes mellitus was the most common comorbidity among patients with infection, followed by chronic kidney disease, chronic obstructive pulmonary disease (COPD), hematological malignancies, and solid cancers. Many previous studies have shown that pulmonary disease is the most frequent comorbidity in patients infected or colonized with S. maltophilia [22,24,27,31,32]. The design of our study does not allow us to compare the risk factors in terms of their predisposition to clinical infection. However, we can clearly say that patients with higher CCI are at higher risk of infection. Recent chemotherapy, immunosuppressant, or steroid use are also associated with an increased risk of infection. The CCI score can be positively correlated with the mortality rate in patients with infection. Our study has also evaluated the use of the IBMP-10 score [33] to predict mortality in patients with ventilator-associated pneumonia (VAP) due to S. maltophilia. An IBMP-10 score of more than 1 has a high risk of mortality in VAP due to S. maltophilia.

Another essential objective of our study was to decide when to start antibiotics in patients with isolates of S. maltophilia. S. maltophilia infections should be treated with antibiotics promptly given that delay inappropriate treatment can contribute to significant mortality. Whether isolating S. maltophilia from a clinical specimen represents true infection rather than colonization warrants careful assessment, as colonization should not be treated, and inappropriate antibiotic use contributes to added adverse effects and the emergence of resistant organisms. In patients with respiratory isolates of S. maltophilia, we recommend initiating appropriate antibiotics in all patients with evidence of infection as per CDCs Specific Site Algorithms for Clinically Defined Pneumonia (PNU 1, 2, and 3) [15]. Patients with S. maltophilia isolates from sterile sites such as blood, CSF, pleural, and peritoneal fluids should also be considered infection rather than colonization, and appropriate antibiotics should be initiated. Patients with CCI ≥ 3 and IBMP-10 score ≥ 2 should be considered for antibiotic therapy as they are at high risk of mortality due to S. maltophilia pneumonia. S. maltophilia is a multidrug-resistant organism, so limited antibiotic options and clinical data. Trimethoprim-sulfamethoxazole (TMP-SMX) has the most reliable in vitro activity against S. maltophilia [34-36]. The drug of choice for S. maltophilia infections is TMP-SMX unless resistance is identified. Only 2.2% of samples in our study were resistant to TMP-SMX (Figure 2). TMP-SMX is administered as 15 mg/kg/day of the TMP component in three or four divided doses, adjusted for renal function [37]. Some patients cannot tolerate TMP-SMX due to hypersensitivity, drug toxicity, or other adverse reactions. In vitro resistance to TMP-SMX among S. maltophilia isolates has been reported in patients with cystic fibrosis [12,13,38,39,40]. The current study did not include the factors associated with in vitro TMP-SMX resistance among S. maltophilia samples. However, this can be studied in a secondary evaluation of existing data from our study. There have been many studies related to the antibiotic susceptibility pattern of S. maltophilia [12,13]. Levofloxacin is a potential alternative to TMP-SMX [34,35]. 73.2% of the isolates from Qatar were susceptible to levofloxacin (Figure 2). We do not recommend using Ciprofloxacin as our study has shown that isolates from Qatar have a high degree of resistance to it. There are no Clinical and Laboratory Standards Institute (CLSI) defined breakpoints for moxifloxacin, even though in vitro studies have shown susceptibility [41]. Hence, among fluoroquinolones, levofloxacin may be used as an alternative to TMP-SMX to treat respiratory infections due to S. maltophilia in Qatar. In our study, Colistin showed excellent in vitro activity against S. maltophilia, with 85% of the samples being sensitive. There is limited international data about the use of colistin S. maltophilia infections. Even though minocycline and tigecycline have low minimal inhibitory concentrations among S. maltophilia isolates [34,35,42] and some studies have shown clinical outcomes comparable to TMP-SMX [43], 65% of the isolates in our study had a tigecycline MIC of ≥ 4 μg/ml. They were considered non-susceptible using interpretive criteria for Enterobacteriaceae [9,44]. There are no MIC interpretive criteria for tetracycline or tigecycline. Also, low serum drug levels and high mortality compared with other pneumonia treatment agents preclude tigecycline use in S. maltophilia associated respiratory and bloodstream infections. Like other international studies, resistance rates to ceftazidime were high in our study (61%) [35]. Only 20 percent of isolates resistant to TMP-SMX and levofloxacin in one study were susceptible to ceftazidime-avibactam, using CLSI breakpoints for ceftazidime alone [45]. S. maltophilia has high resistance rates, either through intrinsic or acquired mechanisms, to other beta-lactams, aztreonam, aminoglycosides, fosfomycin, and carbapenems. Resistance to carbapenems should be presumed regardless of susceptibility testing results [44,45].

Duration of therapy should be seven days for hospital-acquired pneumonia in an immunocompetent host and 10 to 14 days in immunocompromised hosts, with evidence of clinical improvement [46]. Infection control measures such as appropriate use of antibiotics, avoidance of prolonged or unnecessary use of foreign devices, and adherence to hand hygiene practices are important to minimize the incidence of S. maltophilia infections and for reducing the emergence of resistant strains [47]

Limitations

The retrospective observational design of the study limits the data interpretation, as the criteria used by the treating physicians for the use and selection of antibiotics were not clearly defined. Our study design cannot identify whether antibiotics provide mortality benefit in patients with clinical infection due to S. maltophilia.

## Conclusions

S. maltophilia is a frequent nosocomial colonizer, but it can cause nosocomial pneumonia in almost one-third of cases, specifically in immunocompromised and patients with CCI ≥ 3 with a high risk of mortality mechanically ventilated patients with IBMP-10 ≥ 2. Prolonged hospital stay is a risk factor for colonization by S. maltophilia. At the same time, recent chemotherapy, immunosuppressant, or steroid use is a risk factor for hospital-acquired pneumonia due to S. maltophilia. Strains from Qatar show good susceptibility to trimethoprim-sulfamethoxazole (TMP-SMX), colistin, and levofloxacin. TMP-SMX and levofloxacin are the only reliable agents for monotherapy of respiratory infections due to S. maltophilia in Qatar. If the isolate is susceptible to TMP-SMX but not to levofloxacin, we recommend rapid desensitization for patients with an immunoglobulin E mediated hypersensitivity reaction to TMP-SMX. In case of resistance to TMP-SMX and levofloxacin, combination therapy may be used for which an ideal regime is yet to be established.
